# Morphology and Phylogeny of Lyophylloid Mushrooms in China with Description of Four New Species

**DOI:** 10.3390/jof9010077

**Published:** 2023-01-05

**Authors:** Shu-Wei Wei, Bo-Yu Lu, Yang Wang, Wen-Jun Dou, Qi Wang, Yu Li

**Affiliations:** 1 Engineering Research Center of Chinese Ministry of Education for Edible and Medicinal Fungi, Jilin Agricultural University, Changchun 130118, China; 2 College of Plant Protection, Shenyang Agricultural University, Shenyang 110866, China

**Keywords:** four new taxa, Lyophyllaceae, nLSU + ITS, phylogenetic analyses, taxonomy

## Abstract

The lyophylloid agarics are a group of ecologically highly diversified macrofungi, some of which are very popular edible mushrooms. However, we know little about lyophylloid species diversity in China. In this study, we described four new species from China: *Lyophyllum atrofuscum*, *L*. *subalpinarum*, *L. subdecastes*, and *Ossicaulis sichuanensis*. We conducted molecular phylogenetic analyses of Lyophyllaceae based on the nuclear ribosomal RNA gene (nLSU) and the internal transcribed spacer regions (ITS). Phylogenetic analyses by the maximum likelihood method and Bayesian inference showed that the four new species are unique monophyletic species. A key to the species of *Lyophyllum* from China and a key to *Ossicaulis* worldwide were given.

## 1. Introduction

While the importance of fungi as mutualists, decomposers, and pathogens is undisputed, researchers are just beginning to unravel the processes that shape their global species richness and distribution. Previous studies established the monophyly of Lyophyllaceae Jülich and positioned the family within the Tricholomatoid clade, and then Alvarado et al. revealed that Lyophyllaceae may be a putative wider concept or the existence of multiple lineages that are basal to it [1,2,3]. Several new genera established in the past decade are expected to reorganize the system, including *Australocybe* T.J. Baroni, N. Fechner & van de Peppel, *Calocybella* Vizzini, Consiglio & Setti, *Clitolyophyllum* E. Sesli, Vizzini & Contu, *Myochromella* V. Hofst., Clémençon, Moncalvo & Redhead, *Phaeotephrocybe* T.J. Baroni, T.W. Kuyper & van de Peppel, *Nigrocarnea* P. Sparre & Læssøe, *Praearthromyces* T.J. Baroni, T.W. Kuyper & van de Peppel, *Sagaranella* V. Hofst., Clémençon, Moncalvo & Redhead, etc. [1,4,5]. In addition, some species of *Lyophyllum*, *Calocybe* Kühner, *Hypsizygus* Singer, and *Termitomyces* R. Heim have edible value [6], and a few species have medicinal and significant economic importance [7,8,9,10]. In particular, *Lyophyllum shimeji* (Kawam.) Hongo has been recognized as a delicacy, and its price is second only to *Tricholoma matsutake* (S. Ito & S. Imai) Singer in Japan.

Lyophyllaceae has a worldwide distribution [11,12,13], with more than 200 species [14]. The unique characteristic that delimits Lyophyllaceae is the presence of siderophilous granulation in the basidia [15]. *Lyophyllum* P. Karst., is a type genus of Lyophyllaceae, and more than 40 species within this genus. The morphological classifications of Singer [16] were inconsistent with the molecular phylogenetic relationships of *Lyophyllum* [17,18], which explains the reason why some species of *Lyophyllum* used to be easily confused with *Calocybe* and *Tephrocybe* Donk [4,19]. The genus of *Ossicaulis* Redhead & Ginns was erected in 1985, with a north temperate distribution, and four species are known worldwide [20]. It is mainly characterized by adnate, sub-decurrent, or lamellae centrally adnexed to the eccentric stipe, tiny ellipsoid spores, and the presence of clamp connections [21].

Recently, several species of Lyophyllaceae have also been reported in China [22,23,24]. The genus *Lyophyllum* is represented by 14 species in China: *L. decastes* (Fr.) Singer, *L. fumosum* (Pers.) P.D. Orton, *L. transforme* (Lapl.) Singer, *L. trigonosporum* (Bres.) Kühner, *L. loricatum* (Fr.) Kühner, *L. macrosporum* Singer, *L. semitale* (Fr.) Kühner, *L. shimeji*, *L. infumatum* (Bres.) Kühner, *L. immundum* (Berk.) Kühner, *L. pulvis-horrei* E. Ludw. & Koeck, *L. pusillum* Clémençon & A.H. Sm., *L. rhombisporum* Shu H. Li & Y.C. Zhao, and *L. sykosporum* Hongo & Clémençon [25,26,27,28]. Similarly, the genus *Ossicaulis* is represented with two species in China: *Ossicaulis lignatilis* (Pers.) Redhead & Ginns and *Ossicaulis yunnanensis* L.P. Tang, N.K. Zeng & S.D. Yang [29,30].

During our macrofungal exploration of southwestern and northwestern China, we encountered the collections of Lyophyllaceae. Upon further morphological examination of the basidiomata and phylogenetic analyses of the internal transcribed space (ITS) and a larger subunit of the nuclear rDNA (28S), these collections presumably represent three new species of *Lyophyllum* and one new species of *Ossicaulis*, which are described in detail. Furthermore, the species of *Lyophyllum* in China are compared in detail, and a worldwide key for *Ossicaulis* is given.

## 2. Materials and Methods

### 2.1. Specimen Sampling

All the specimens used in this study were collected in 2018–2021. These samples were dried overnight using an electric oven at 40 °C and deposited in the Herbarium Mycology of Jilin Agricultural University (HMJAU).

### 2.2. Morphological Observation

The macro-morphological descriptions were recorded in the field, and images of the basidiocarps were taken in the field with an OLYMPUS E-P7. The color code and terminology followed Kornerup and Wanscher [31]. Tiny tissue was cut from the dried basidiomata using a sharp blade, and micro-morphological structures were observed via a light microscope (ZEISS Axioscope 5, ZEISS, Jena, Thuringia, Germany) performed in 5% KOH solution and then in Melzer’s reagent solution or Acetcoarmine solution. Twenty basidiospores and basidia were measured from each specimen. Dimensions are given as (a)b–c(d), of which ‘b–c’ refers to the minimum of 90% of the measured values, and a or d represents the extreme values. Factor Q refers to the aspect ratio of each basidiospore in the side view; Lm/Wm defines the average length/width of all measured basidiospores ± sample standard deviation.

### 2.3. DNA Extraction, Amplification, and Sequencing

Total genomic DNA was extracted using the Plant Genomic DNA Kit (Tiangen Biotech Co., Ltd., Beijing, China). The nuclear ribosomal internal transcribed spacer (ITS) and nuclear ribosomal large subunit (nLSU) sequences were amplified using primer pairs of ITS4/ITS5 and LR0R/LR5, respectively [32,33,34]. The reactions were performed with the following program: initial denaturation at 95 °C for 4 min (ITS) or 3 min (nLSU), 35 cycles at 95 °C for 40 s, 58 °C (ITS) for 40 s or 52 °C (nLSU), and 72 °C for 80 s (ITS) or 120 s (nLSU); for terminal elongation the reaction batches were incubated at 72 °C for 10 min. Then, PCR productions were sent to Sangon Biotech Co., Ltd. (Shanghai, China) to be directly sequenced using an ABI 3730*xl* DNA analyzer.

### 2.4. Phylogenetic Analyses

The newly generated sequences in this study have been deposited in GenBank (https://www.ncbi.nlm.nih.gov/genbank/, accessed on 10 October 2022), with other similar sequences downloaded from the NCBI (https://www.ncbi.nlm.nih.gov/, accessed on 10 October 2022) datasets, and *Entoloma undatum* (Gillet) M.M. Moser as the outgroup. For the datasets (see Table 1), the alignment was generated for ITS and nLSU datasets using the “L–INS–i” strategy of MAFFT v.7.017 [35]. Before performing phylogenetic analyses, start and end ambiguous sites were removed, and gaps were manually adjusted to optimize the alignment by BioEdit v7.1.3 [36] and then were combined by Phylosuite v1.2.2 [37]. The best-fit evolutionary model was estimated by using Modelfinder [38]. Phylogenetic analyses were carried out using the Bayes inference (BI) and maximum likelihood (ML). BI analysis using Markov chain Monte Carlo (MCMC) methods were carried out with MrBayes 3.2.6 [39], running in 2,000,000 generations, and sampled every 1000 generations. The initial 25% of the sampled data were discarded as burn-in, other parameters were kept at the default settings. For ML analysis, the datasets were analyzed using IQ-TREE under an ultrafast bootstrap, with 5000 replicates [40]. The posterior probability ≥ 0.95 for Bayesian inference analysis (BI-PP) and bootstrap proportions ≥ 70% for ML analysis (ML-BP) were considered significant values. Trees were edited using FigTree version 1.4.4 (http://tree.bio.ed.ac.uk/software/figtree/, accessed on 10 October 2022).

## 3. Results

### 3.1. Molecular Phylogeny

**Figure 1:**
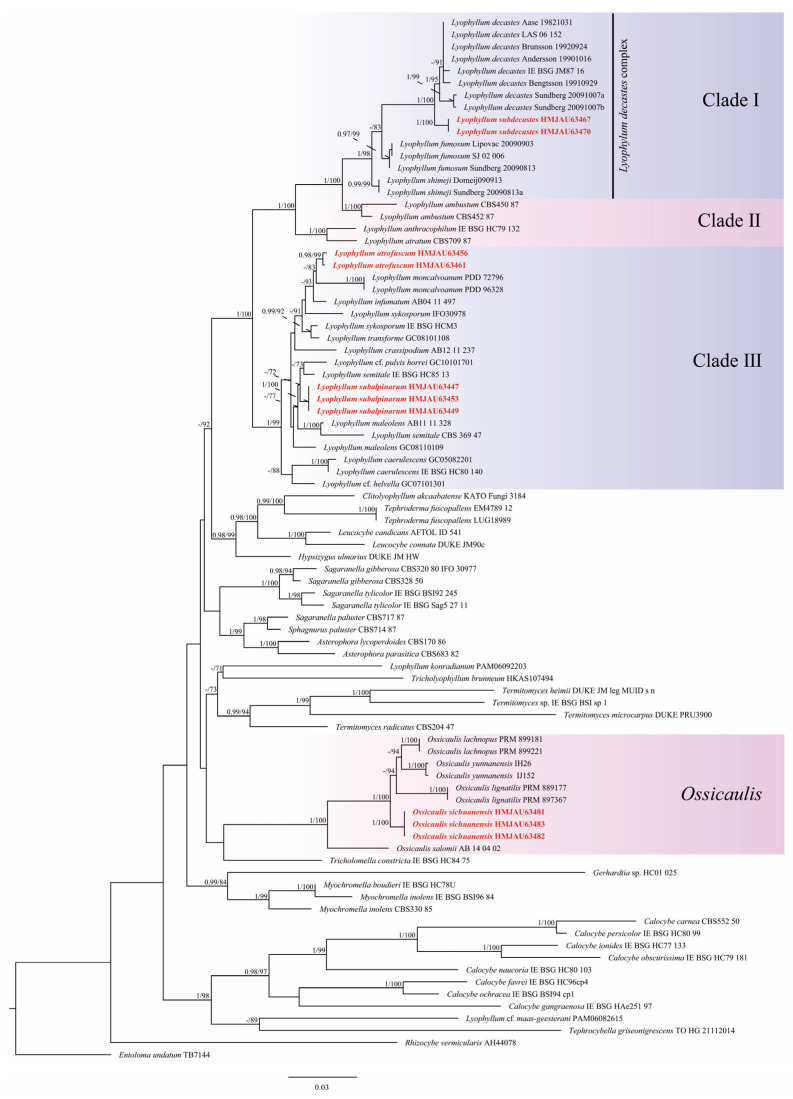
Phylogenetic tree performed by ML analysis based on ITS + nLSU sequences. Branches are labeled with Bayesian posterior probabilities ≥ 0.95 and ML bootstrap values ≥ 70%. The new species are indicated in red.

Fourteen sequences were newly generated from specimens of *Lyophyllum*, and six sequences were newly generated from specimens of *Ossicaulis*, all of which were collected from China and deposited in GenBank (Table 1). A combined dataset of two markers, including 1616 bases, was used to execute the BI and ML analyses. Amongst the dataset, 841 were constant sites, 224 were variable and parsimony-uninformative sites, and 551 were parsimony-informative sites. Based on the Bayesian information criterion (BIC), the GTR + F + I + G4 models were selected as the substitution model for the ITS and nLSU partitions. The phylogenetic construction performed by the BI and ML analyses showed a similar topology. Therefore, we selected the ML tree as the working phylogenetic hyphothesis, with the Bayesian posterior probabilities ≥0.95 and ML bootstrap values ≥ 70% labeled along the branches (Figure 1). In the phylogram, *L. subdecastes*, *L. decastes*, *L. fumosum*, and *L. shimeji* were grouped in Clade Ⅰ (sect. *Difformia* (Singer)). *Lyophyllum ambustum* (Fr.) Singer, *L. anthracophilum* (Lasch) M. Lange & Sivertsen, and *L. atratum* (Fr.) Singer were grouped in Clade Ⅱ. *Lyophyllum atrofuscum* related to *L. moncalvoanum* was grouped in Clade Ⅲ (sect. *Lyophyllum* (Singer)) with weak support. *Lyophyllum subalpinarum* related to *L.* cf. *pulvis-horrei* and *L. semitale* grouped were in Clade Ⅲ (sect. *Lyophyllum* (Singer)) showed weak support. In addition, *Ossicaulis sichuanensis* formed a clade with *O. lachnopus* (Fr.) Contu, *O. lignatilis* (Pers.) Redhead & Ginns, *O. salomii* Siquier & Bellanger, and *O. yunnanensis*.

### 3.2. Taxonomy

**Figure 2:**
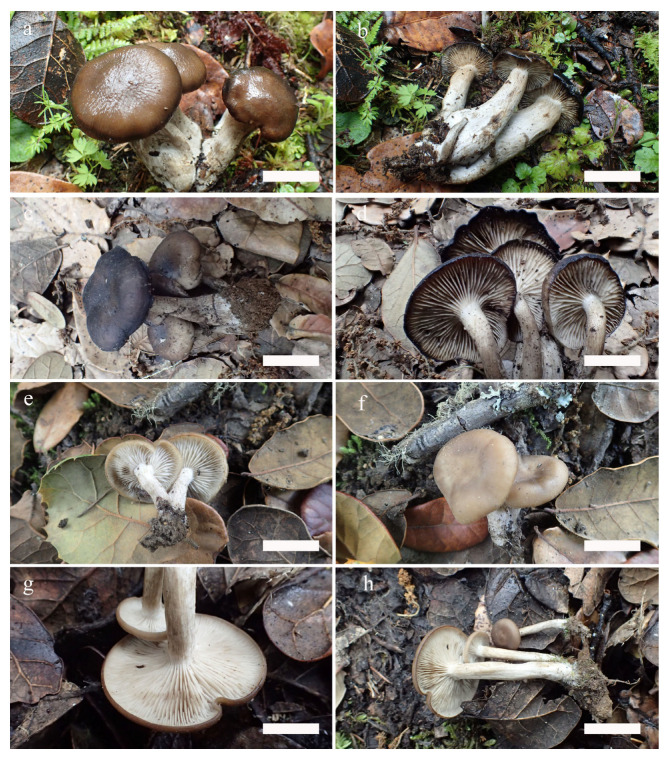
Basidiomata of *Lyophyllum*. (**a**–**d**) *L. atrofuscum*, (**a**,**b**) HMJAU63461; (**c**,**d**) HMJAU63456! holotype. (**e**–**h**) *L. subalpinarum,* (**e**,**f**) HMJAU63447! holotype; (**g**,**h**) HMJAU63453. Scale bars: (**a**,**d**) 4 cm; (**b**) 5 cm; (**c**) 3 cm; (**e**–**g**) 2 cm; (**h**) 3 cm.

**Figure 3:**
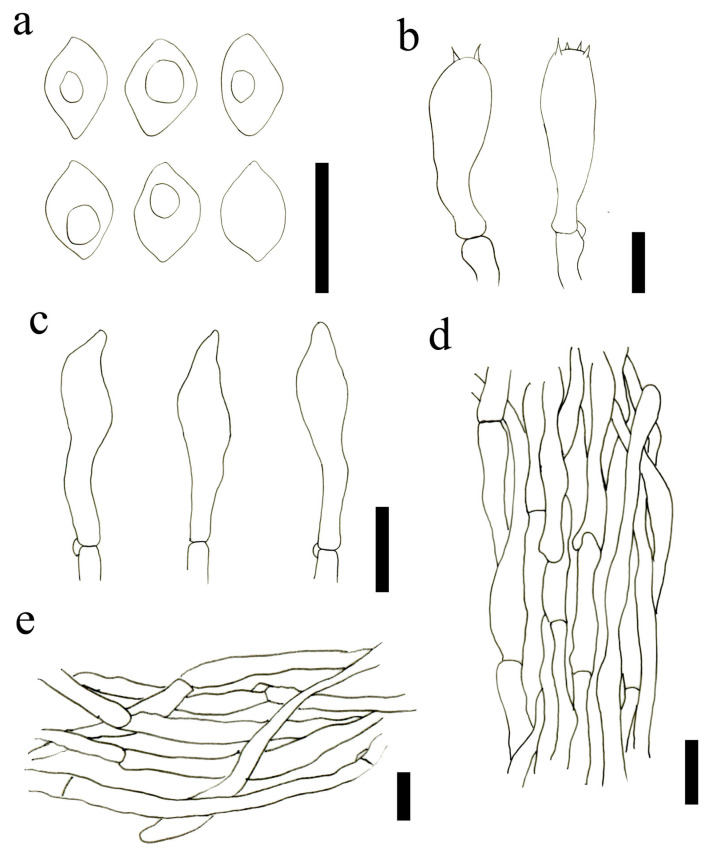
Microscopic features of *L. atrofuscum* (HMJAU63460). (**a**) Basidiospores; (**b**) Basidia; (**c**) Pleurocystidia; (**d**) Stipitipellis; (**e**) Pileipellis. Scale bars: (**a**–**d**) = 10 μm; (**e**) = 15 μm.

*Lyophyllum atrofuscum* S.W. Wei, Q. Wang & Y. Li, sp. nov. (Figure 2a–d and Figure 3)

MycoBank number. MB846029

Etymology. The specific name *atrofuscum* (Latin). “ater” refers to the black, sable, dark, gloomy; “fuscus” refers to the swarthy, dusky, dark.

Holotype. China, Xizang Autonomous Region, Lingzhi Prefecture, Lulang Town, Jiagapu, 29°40′ N, 94°43′ E, alt. 3664 m, 12 August 2021, Shu-Wei Wei (HMJAU63456!, ITS = OP605494, 28S = OP605514).

Pileus 2.5–9.0 cm broad, hemispherical to broadly convex when young, becoming plane to plano-concave, often with depressed, center and in-rolled margin when mature, light brown (6D4), yellowish-brown (5D8), brownish-orange (7C3), to grayish-brown (7D3), dark at the center. Sometimes margin wavy, pileus context watery soaked in wet weather conditions, brown (7E8) or reddish-brown (8D6). It was staining bluish-gray to black immediately when broken. Context white, up to 0.5 cm thick at the pileus center, is thinner toward its margin and discolored when exposed or injured. Lamellae adnate to slightly decurrent, moderately broad, tapering toward the margin white at first, becoming yellowish-white (4A2), yellowish-white (3A2), to brownish-gray (6C3) with age, usually discoloring to dark at the edge when touched or injured, with 1–3 unequal lamellulae between two entire lamellae. Stipe 3.0–10.0 cm long, 0.7–1.5 cm thick, cylindrical to clavate, usually equal, occasionally enlarged at the base, white at first, grayish-white (6B1) with brownish-gray (6C2) when mature, usually paler than the pileus, sometimes nearly black in the upper, scarcely darkening below, longitudinally fibrillose, base white-mycelioid. Flour flavor.

Basidiospores (5.0)5.5–7(7.5) × 3.9–5.0(5.5) μm, Lm = 6.38 ± 0.67, Wm = 4.67 ± 0.48, Q = (1.1)1.2–1.5(1.7), Qm = 1.37 ± 0.13, ellipsoid, subrhomboid to irregular rhomboid, smooth, hyaline, thin-walled. Basidia mainly two–four spored, (27.3)27.5–36.5(37.0) × (6.8)7.5–10.0(10.5) μm, Q = (2.8)3.0–10.0(10.5), Qm = 4.04 ± 0.49, clavate, siderophilous granules abundant, some with basal clamp connections. Subhymenium is made up of inflated hyaline elements. Hymenophoral trama is regular, hyaline hyphae, made up of thin. Pleurocystidia scattered, 18.96–28.55 × 4.48–6.93 μm, long fusiform, narrowly lageniform to irregular, with subacute apex or long beak, thin-walled. Cheilocystidia not observed. Pileipellis composed of 4.0–9.5 μm wide hyphae, cylindrical hyphae, smooth, with intraparietal pigment. Stipitipellis arranged regular, hyphae parallel, not constricted at the septa, smooth, composed of 2.5–6.0 μm wide hyphae. Clamp connections are present.

Known distribution. Known to occur in the subalpine regions at high elevations (usually above alt. 3000 m) in Sichuan, Tibet, and Yunnan of Southwest China.

Habit and habitat. Scattered to gregarious on soil in forests dominated by *Quercus semecarpifolia*, from August to October.

Additional specimens examined. China. Sichuan Province: Ganzi Prefecture, Jiulong County, 29°5′ N, 101°23′ E, alt. 3375 m, 25 August 2020, Shu-Wei Wei (HMJAU63457); Jiulong County, Wuxuhai, 29°3′ N, 101°24′ E, alt. 3198 m, 26 August 2020, Shu-Wei Wei (HMJAU63458); Jiulong County, Jishoushan, 29°5′ N, 101°23′ E, alt. 3340 m, 23 August 2021, Shu-Wei Wei (HMJAU63459), Shu-Wei Wei (HMJAU63460). Xizang Autonomous Region: Lingzhi Prefecture, Lulang Town, Jiagapu, 29°40′ N, 94°43′ E, alt. 3664 m, 12 August 2021, Shu-Wei Wei (HMJAU63461); Lingzhi Prefecture, Lulang Town, Gongcuo Lake, 29°45′ N, 94°44′ E, alt. 3368 m, Shu-Wei Wei (HMJAU63462). Yunnan Province: Shangri-la City, Xiaozhongdian, 27°24′ N, 99°49′ E, alt. 3464 m, 29 August 2021, Shu-Wei Wei (HMJAU63463), Shu-Wei Wei (HMJAU63464), Shu-Wei Wei (HMJAU63465), Shu-Wei Wei (HMJAU63466).

Notes: Compared with the species with dark brown to fuscous pileus and black when bruised of the lamellae, *L. bonii* Contu*, L. fuscobrunneum* Dähncke, Contu & Vizzini, *L. rhombisporum*, and *L. solidipes* Clémençon & A.H.Sm. are similar to the new species. *L. bonii* from the Canary Islands, differ by having more narrower stipe, elongate and stout basidia (exceeding 35 μm) [48]. *L. fuscobrunneum* from the Canary Islands differ by having the undertint pileus, narrow hyphae in stipitipellis, and longer basidia [49]. *L. solidipes* from Hood National Forest of the United States has narrower hyphae of stipitipellis (3–5 μm) and narrower basidiospores (Q = 1.5–1.9) [50]. *L. rhombisporum* from China is characterized by rhombic or subrhombic basidiospores (14.5–17.0 × 10.0–11.5 μm) and larger basidia (36.5–46.0 × 10.0–11.3 μm) [51].

Phylogenetic analyses suggest that the new species is closely related to *L. infumatum*, *L. moncalvoanum*, and *L. sykosporum*. The common feature is that the context and lamellae turn black after injury. *Lyophyllum infumatum* from Italy is bigger in size of the basidiospore (9.2–12.2 × 5.0–7.4 μm), and the hyphae of pileipellis is 1.0–2.0 μm wide. *Lyophyllum sykosporum* from Japan and Switzerland is characterized by triangular basidiospores (5.5–8.5 × 4.5–6.5 μm), whitish and pruinose toward the stipe apex [52]. *Lyophyllum moncalvoanum* from New Zealand is characterized by olivaceous black pileus, clay to the olivaceous stipe, and globose basidiospores (5.0 ± 0.5 μm) [44].

**Figure 4:**
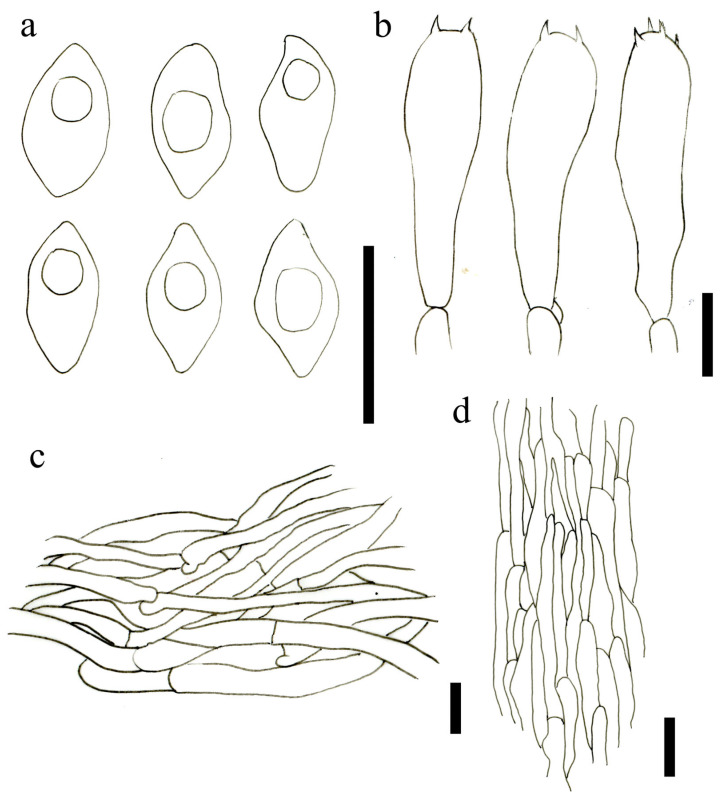
Microscopic features of *L. subalpinarum* (HMJAU63451). (**a**) Basidiospores; (**b**) Basidia; (**c**) Pileipellis; (**d**). Stipitipellis. Scale bars: (**a**,**b**) = 10 μm; (**c**,**d**) = 20 μm.

*Lyophyllum subalpinarum* S.W. Wei, Q. Wang & Y. Li, sp. nov. (Figure 2e–h and Figure 4)

MycoBank number. MB846028 

Etymology. “*subalpinarum*”, the area near alpine region.

Holotype. China, Xizang Autonomous Region, Lingzhi Prefecture, Lulang Town, Gongcuo Lake, 29°45′ N, 94°44′ E, alt. 3368 m, 20 August 2020, Shu-Wei Wei (HMJAU63447!, ITS = OP605492, 28S = OP605512).

Pileus 2.5–6.0 cm, hemispherical to convex with an inrolled margin when young, expanding to broadly convex or plane, shallowly depressed when mature, dry, glabrous, grayish-yellow (4C4), honey-yellow (4D6), brownish-orange (5C5), to yellowish-brown (5D5), darker at the center, brownish-red (8C8) to reddish-brown (8E8) when soaked in wet weather conditions. Context 0.3–0.7 cm, white to yellowish-white, dark when exposed or injured. Lamellae bluntly adnate to subdecurrent, moderately close, tapering toward the margin, white at first, becoming yellowish-white (4A2), orange-white (5A2) to grayish-orange (6B3) with age, usually discoloring to black at the edge when touched or injured, slight, with 1–3 unequal lamellulae between two entire lamellae. Stipe 2.5–7.5 cm long, 0.5–1.0 cm thick, equal or attenuate at the base, hollow, pliant, surface whitish-gray, dark color in the middle, obscurely longitudinally striate, not noticeably discoloring where bruised.

Basidiospores (6.7)6.9–8.7(9.4) × (4.0)4.3–5.1(5.6) μm, Lm = 7.91 ± 0.71, Wm = 4.75 ± 0.40, Q = (1.3)1.5–1.9(2.0), Qm = 1.67 ± 0.13, rounded-cylindrical to an irregular rhombus, mostly uninucleate, more rarely binucleate, smooth, thin-walled. Basidia (30.1)30.6–36.7(38.2) × (7.1)7.4–8.9(9.5) μm, Q = (3.5)3.8–4.7(5.0), Qm = 4.27 ± 0.41, four spored, rarely two-spored, siderophilous granules abundant, some with basal clamp connections. Pleurocystidia and cheilocystidia not observed. Pileipellis is a cutis of parallel to interwoven cylindrical hyphae, smooth, cylindrical, thin-walled, composed of 3.0–9.0 μm wide hyphae. Stipitipellis arranged regularly, parallel, cylindrical, composed of 3.0–7.5 μm wide hyphae. Clamp connections are present.

Known distribution. Known to occur in the Xizang Autonomous Region at high elevations (usually above alt. 3000 m) in Southwest China.

Habit and habitat. Single to scattered on soil in forests dominated by Spruce forest, from August to October.

Additional specimens examined. China. Xizang Autonomous Region: Lingzhi Prefecture, Bayi District, Katian Village, 29°44′ N, 94°10′ E, alt. 3057 m, 10 August 2020, Shu-Wei Wei (HMJAU63448), Shu-Wei Wei (HMJAU63449); Lingzhi Prefecture, Bomi County, Guxiang Village, 29°54′ N, 95°26′ E, alt. 3230 m, 16 August 2020, Shu-Wei Wei (HMJAU63450), Shu-Wei Wei (HMJAU63451); Lingzhi Prefecture, Lulang Town, Gongcuo Lake, 29°45′ N, 94°44′ E, alt. 3368 m, 20 August 2020, Shu-Wei Wei (HMJAU63452); Lingzhi Prefecture, Ladingga Village, 29°38′ N, 94°23′ E, alt. 3283 m, 21 August 2020, Shu-Wei Wei (HMJAU63453), Shu-Wei Wei (HMJAU63454), Shu-Wei Wei (HMJAU63455).

Notes: Morphologically, the paled-colored stipe and basidiospores size of *L. subalpinarum* is strongly reminiscent of *L. deliberatum* (Britzelm.) Kreisel, *L. aemiliae* Consiglio, *L. pallidum* Clémençon & A.H. Sm, *L. canescentipes* Clémençon & A.H. Sm, and *L. fistulosum* Clémençon & A.H. Sm. However, *L. deliberatum* has broader pileus (3.0–9.0 cm) and longer basidiospores (8.5–11.5 × 5–6.5 μm) [13]; and *L. aemiliae* differs by having white, pale gray to orange and Emarginate lamellae [13]. *Lyophyllum pallidum* from the United States differs by having smaller and wider basidiospores (6.2–7.4 × 4.5–5.2 μm, Q = 1.3–1.5) and pale watery-gray pileus. The main characteristics of the pileus of *L. canescentipes* are brownish-gray with the faintly striate margin, wider basidiospore (6.9–9.1 × 4.7–6.4 μm), and longer basidia (36–40 × 7–8 μm) [50]. The dark fuliginous pileus of *L. fistulosum* could be significantly different from this species [50].

Phylogenetically, the new species is closely related to *L. semitale* and *L. maleolens*. However, *L. semitale* from Korea and North Carolina (in the United States) is bigger in size regarding the basidiospore (3.0-8.0 cm); at the same time, darker-colored pileus and pale gray to brownish-gray lamellae also help to distinguish it from the new species [53,54]. Additionally, *L. maleolens* from Spain is characterized by a brown to dark fuscous-brown pileus, and the stipe is wider than that of the new species [48].

**Figure 5:**
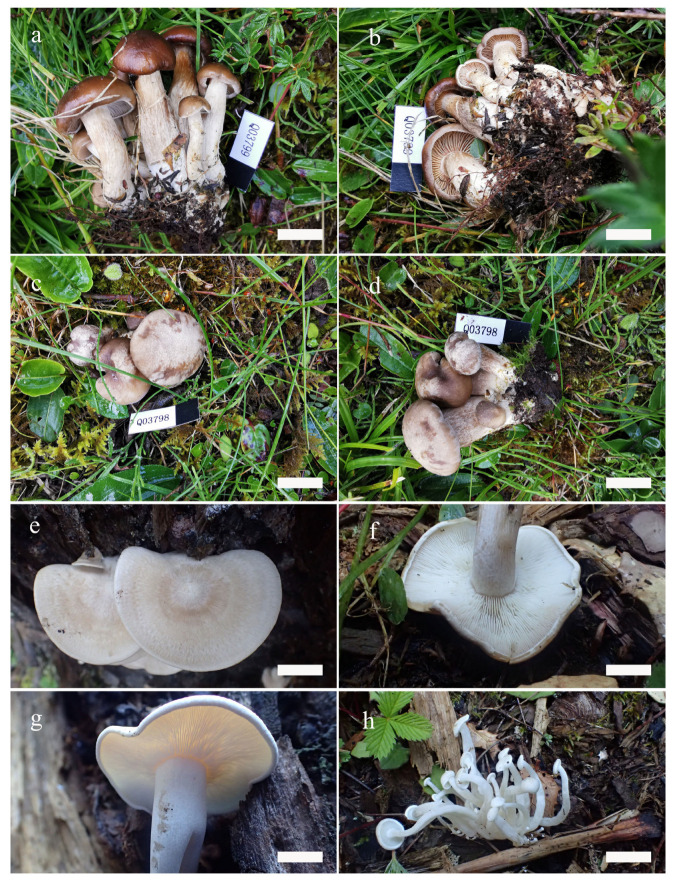
Basidiomata of *Lyophyllum*. (**a**–**d**) *L. subdecastes*, (**a**,**b**) HMJAU63467! holotype; (**c**,**d**) HMJAU63470. Basidiomata of *Ossicaulis*. (**e**–**h**) *O. sichuanensis*, (**e**–**g**) HMJAU63480! holotype; (**h**) HMJAU63483. Scale bar: (**a**–**d**) 2 cm; (**e**,**f**) 1.6 cm; (**g**) 1.5 cm; (**h**) 2 cm.

**Figure 6:**
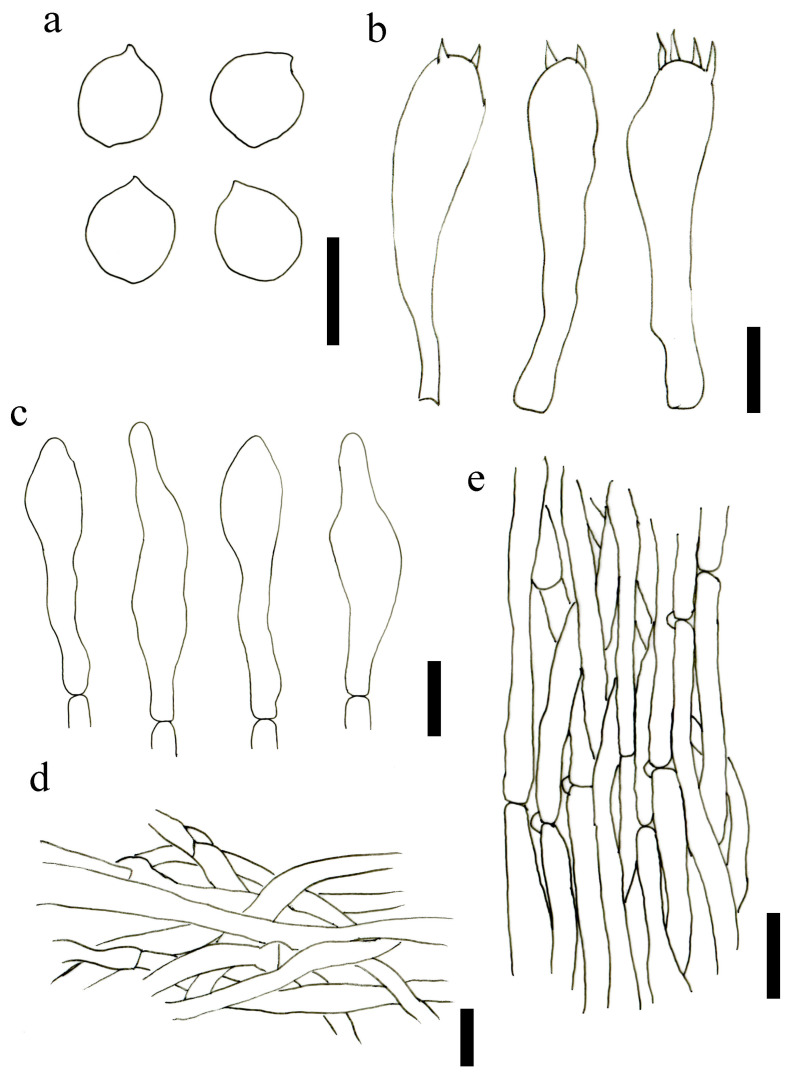
Microscopic features of *L. subdecastes* (HMJAU63468). (**a**) Basidiospores; (**b**) Basidia; (**c**) Pleurocystidia; (**d**) Pileipellis; (**e**) Stipitipellis. Scale bars: (**a**) = 5 μm; (**b**–**e**) = 10 μm.

*Lyophyllum subdecastes* S.W. Wei, Q. Wang & Y. Li, sp. nov. (Figure 5a–d and Figure 6)

MycoBank number. MB846030

Etymology. “sub” means “near”, named because it is similar to *L. decastes*.

Holotype. China, Gansu Province, Zhangye City, Kangle grassland, 38°47′ N, 99°47′ E, alt. 2793 m, 08 August 2019, Shu-Wei Wei & Bo-Yu Lu (HMJAU63467!, ITS = OP605489, 28S = OP605509).

Plieus 2.5–5.5 cm wide, hemispherical to convex when young, broadly convex with an inrolled margin, elastic-cartilaginous, variable in shape; according to growth conditions, flatter when mature, without umbo, dry, glabrous, and sometimes margin wary, yellowish-brown (5E8), brown (6E6), grayish-red (8B5), dark at center, paler toward the margin, orange-white (5A2) at the margin of some young pileus. Significant deepening to reddish-brown (9E8) or brownish-red (10D8) when soaked in wet weather conditions. Context 0.4–1.3 cm thick, white to pale-white, fleshy, unchanging when exposed or injured, white in exsiccate. Lamellae close, adnate to subdecurrent, white at first, becoming yellowish-white (4A2) with age. Stipe 2.7–6.6 cm long, 0.5–1.5 cm thick, cylindrical to clavate, fibrillose-striate, base often enlarged when young, and usually equal over time, white at first, orange-white (6A2), with reddish-gray (7B2) to grayish-red (8C3) when mature, discoloring slightly when touched or damaged, fleshy and solid inside. Taste mild. Odor indistinctive.

Basidiospores (3.7) 3.9–5.0(5.3) × (3.6)3.7–5.0(5.2) μm, Lm = 4.47 ± 0.49, Wm = 4.25 ± 0.46, Q = 1.0–1,1, Qm = 1.05 ± 0.03, globular or subglobular, smooth, with a single, central oil-drop, nonamyloid in Melzer’s reagent. Basidia mainly four spored, rarely two spored, (31.8)36.7–50.6(53.5) × (8.0)8.4–10.9(11.1) μm, Q = (3.5)3.7–5.5(6.1), Qm = 4.51 ± 0.57, clavated, thin-walled, sterigmata 2.3–4.8 μm, siderophilous granules abundant. Pleurocystidia scattered, 21.63–47.57 × 5.23–11.19 μm, fusoid-ventricose to broadly fusoid-ventricose, with subacute apex or long beak, thin-walled. Cheilocystidia not observed. Pileipellis is a cutis of parallel to interwoven cylindrical hyphae, smooth, cylindrical, thin-walled, composed of 4.0–8.0 μm wide hyphae. Stipitipellis a cutis of parallel, regular, clamped, smooth, cylindrical, composed of 3.0–7.0 μm wide hyphae. Clamp connections are present.

Known distribution: Known to occur in the subalpine regions at high elevations (usually above alt. 2500m) in the Qilian Mountain, Gansu Province of Northwest China.

Habit and habitat: Gregarious on the soil in the coniferous forest dominated by Qinghai spruce, from August to October.

Additional specimens examined: China. Gansu Province: Zhangye City, Si Dalong tree farm, 37°38′ N, 102°38′ E, alt. 3040 m, 23 August 2018, Shu-Wei Wei & Bo-Yu Lu (HMJAU63468); the same location, in coniferous forest, mainly dragon spruce, 3017 m, 13 August 2019, Shu-Wei Wei & Bo-Yu Lu (HMJAU63478), Shu-Wei Wei & Bo-Yu Lu (HMJAU63479); the same location, in coniferous forest, mainly dragon spruce, 2998 m, 28 September 2018, Shu-Wei Wei & Bo-Yu Lu (HMJAU63469); Xiama tree farm, 37°38′ N, 103°9′ E, alt. 2698 m, 21 July 2019, Shu-Wei Wei & Bo-Yu Lu (HMJAU63470); Kangle grassland, 38°47′ N, 99°47′ E, alt. 2793 m, 08 August 2019, Shu-Wei Wei & Bo-Yu Lu (HMJAU63471), Shu-Wei Wei & Bo-Yu Lu (HMJAU63472), Shu-Wei Wei & Bo-Yu Lu (HMJAU63473), Shu-Wei Wei & Bo-Yu Lu (HMJAU63474), Shu-Wei Wei & Bo-Yu Lu (HMJAU63475), Shu-Wei Wei & Bo-Yu Lu (HMJAU63476), Shu-Wei Wei & Bo-Yu Lu (HMJAU63477).

Notes: The new species can be identified by molecular-phylogenetic, morphological, and ecological characteristics. In the phylogenetic tree (Figure 1), this species forms an independent clade within the *L. decastes* complex and differs from the East Asian and European clade of *L. decastes* and all other clades. In the field (Figure 5a–d), this taxon can be recognized by its yellowish-brown to brown pileus; lamellae close, adnate to subdecurrent; context and stipe fleshy and solid inside. It has a high-yielding production from August to October every year. Ecologically, it is distributed in high-altitude areas in Northwest China, usually over or around an elevation of 3000 m. Its subalpine distribution in Asia helps distinguish it from similar taxa from Europe and the United States.

Following the morphological analyses, *L. subdecastes* should be placed in *L. decastes* complex [55,56]; *L. decastes*, *L. shimeji*, and *L. loricatum* are similar to *L. subdecastes* in their appearance. However, the specimens found in Poland of *L. decastes* differ from *L. subdecastes* in their broader pileus, slightly longer stipe, lower altitude of distribution areas, and slightly larger basidiospores 5.0–7.0 μm [52,57]. *L. shimeji* differs from *L. subdecastes* with a slightly broad pileus, robust stipes, and an inflated base [43,58]. The differences between the new species and *L. loricatum* are that the latter has a reddish-brown to chestnut-brown pileus, which easily removes the pileus epiderm [15,59], and the basidia of specimens in Switzerland are smaller (28.0–32.0 × 7.0–8.0 μm) [52]. The results of phylogenetic analyses suggest that *L. subdecastes* is closely related with *L. decastes*, *L. fumosum*, and *L. shimeji*, which is consistent with the morphological study.

**Figure 7:**
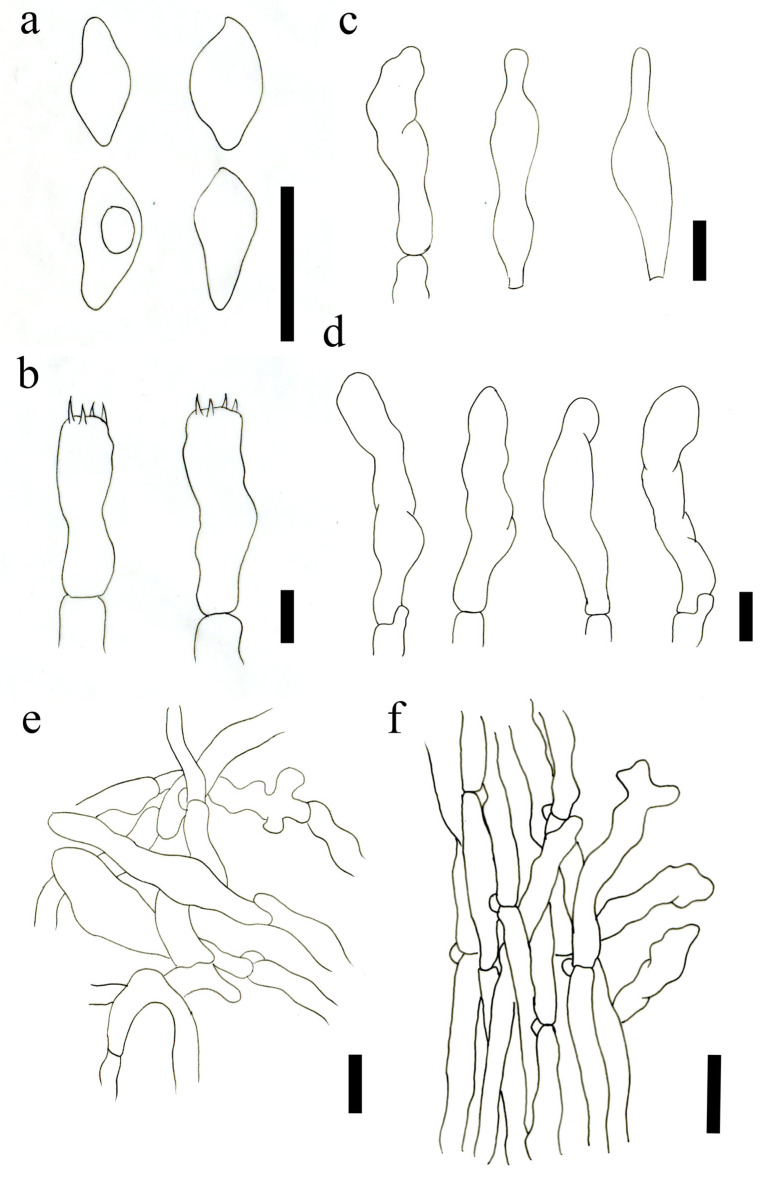
Microscopic features of *O. sichuanensis* (HMJAU63480!, holotype). (**a**) Basidiospores; (**b**) Basidia; (**c**) Cheilocystidia; (**d**) Pleurocystidia; (**e**) Pileipellis; (**f**) Stipitipellis. Scale bars: (**a**–**e**) = 5 μm; (**f**) = 10 μm.

*Ossicaulis sichuanensis* S.W. Wei, Q. Wang & Y. Li, sp. nov. (Figure 5e–h and Figure 7)

MycoBank number. MB846031

Etymology. “*sichuanensis*”, refers to Sichuan Province, China, the holotype locality.

Holotype: China, Sichuan Province: Ganzi Prefecture, Jiulong County, Wuxuhai, 29°3′ N, 101°24′ E, alt. 3207 m, 26 August 2020, Shu-Wei Wei (HMJAU63480!).

Pileus 4.0–6.0 cm wide, shell-shaped to semicircular, applanation to slightly depressed at the center; margin involute or incurved when young, becoming wavy with age, sometimes lobed when mature, surface snow white to chalky (1A1, 2A1, 3A1, 4A1) in immature stages, yellowish-white to orange-white when old (3A2, 4A2, 5A2), cespitose, small to medium-sized, velutinous to tomentous; context 0.4–0.8 cm thick in the center, whiteish to cream-white, progressively thinning toward the margin, hygrophanous and opaque, unchanging in color when bruised. Lamellae free, somewhat crowded, narrow (0.2 cm high), thin, with one–three unequal lamellae between two entire lamellae, white to whiteish (1A1, 2A1, 3A1, 4A1). Stipe 2.6–3.5 cm long, 0.8–1.2 cm thick, central to nearly central when young, eccentric to lateral with age, subcylindrical to cylindrical, slightly wider upwards, surface snow white to whiteish (1A1, 2A1, 3A1, 4A1), finely pubescent, hygrophanous, unchanging when exposed. Odor and smell faint, taste not recorded.

Basidiospores (4.0)4.5–5.5 (6.0) × 2.5–3.0(3.5) μm, Lm = 5.11 ± 0.54, Wm = 2.86 ± 0.28, Q = (1.5)1.6–1.8(2.0), Qm = 1.73 ± 0.26, smooth, obtusely amygdaloid, thin-walled, hyaline. Basidia (15.0)16.5–20.0(22.5) × (4.0)4.5–5.0(6.0) μm, Q = (3.0)3.5–5.6(5.7), Qm = 4.21 ± 0.72, thin-walled, narrow clavate, 4-spored, colorless to hyaline in KOH. Basidiolae 16.0–20.0 × 4.0–5.0 μm, narrowly clavate. Cheilocystidia 12.0–23.0 × 3.0–6.5 μm, thin-walled, flexuous to irregular, cylindrical or subcylindrical, narrowly clavate, hyaline to colorless, rarely diverticulate outgrowths. Pleurocystidia 12.9–27.4 × 2.6–6.0 μm, similar to cheilocystidia in shape. Lamellar trama regular and interweave, 3.0–8.0 μm broad. Pileipellis is a cutis parallel to slightly interwoven cylindrical hyphae, composed of 3.0–6.0 μm wide, thin-walled, hyaline to colorless, terminal cells 11.0–25.0 × 3.5–6.0 μm, narrowly calvate or subcylindrical. Stipitipellis an interwoven, composed of 2.0–5.5 μm wide hyphae, hyaline to colorless in KOH, thin-walled hyphae, terminal cell 16.0–33.0 × 3.0–8.0 μm, cylindrical, narrowly clavate or irregular. Clamp connections are present.

Known distribution: Known to occur in the subalpine regions at high elevations (usually above alt. 3500 m) in Sichuan Province of Southwest China.

Habit and habitat: Cespitose, on living tree trunk dominated by *Rhododendron* spp., from August to October.

Additional specimens examined: China. Sichuan Province: Ganzi Prefecture, Jiulong County, Wuxuhai, 29°3′ N, 101°24′ E, alt. 3207 m, 26 August 2020, Shu-Wei Wei (HMJAU63481), Shu-Wei Wei (HMJAU63482), Shu-Wei Wei (HMJAU63483).

Notes: Amongst the known species within *Ossicaulis* with snow white to chalky pileus and similar lamellae spacing, *O. salomii*, *O. lachnopus*, *O. lignatilis*, and *O. yunnanensis* are close to the new species. However, *O. salomii*, which is from Spain, can be distinguished by its smaller, caramel pileus, habitat in the dune zone next to the sea, and narrow stipe [45]; *O. lachnopus* and *O. lignatilis* from Europe differ by having smaller Basidia (12.0–15.0 × 3.5–4.5 μm), and the gill attachment of *O. lachnopus* is significantly different from that of *O. sichuanensis*. Phylogenetic analyses suggest that *O. lachnopus*, *O. lignatilis*, and *O. yunnanensis* have close affinities with *O. sichuanensis*. Consistent with the morphological study.

## 4. Discussion

To date, only 14 species of *Lyophyllum* have been previously reported in China, and most of them were collected from the temperate continental and plateau mountainous climate areas. In this study, four new species, *L. atrofuscum*, *L. subalpinarum*, *L. subdecastes*, and *O. sichuanensis*, are described from temperate and boreal China, based on morphological studies and phylogenetic analyses.

According to Bellanger et al., species of *Lyophyllum* are mostly distributed in north temperate regions [3]. In the molecular phylogenetic analyses based on the dataset combining ITS and nLSU, *Lyophyllum* and other members of Lyophyllaceae appear to be as a polyphyletic group, which is consistent with previous studies. Hofstetter et al. found that ancestral states of Lyophyllaceae s. str. and s.l. were unequivocally reconstructed as saprotrophic, while parasitism, ectomycorrhiza, and insect association appear to be derived states in the evolution of Lyophyllaceae [1]. The new species described in this study occupy independent lineages in Lyophyllaceae. *Lyophyllum* Clade Ⅰ (sect. *Difformia*) are very similar in morphology. There is also a large amount of interspecific similarity in basidiocarp form, coloration, size, and lamellae attachment. Five taxa recognized in the *L. decastes* complex in Japan and Europe were confirmed by previous research [60,61]. In this study, *L. subdecastes* and *L. decastes* were grouped in a single sister-clade with strong support (1/100%), and they can be distinguished by morphology and phylogeny. *Lyophyllum subdecastes* grows on soil under the subalpine coniferous forests dominated by *Picea crassifolia*, whereas *L. decastes* differs from *L. subdecastes* in a broader pileus, longer stipe, and a main distribution in lower altitude areas [52]. Moncalvo et al. found that the mycelia cultural characters of *L. atratum* were closer to Clade Ⅰ (sect. *Difformia*) [62]. In this study, we failed to obtain the specimens and corresponding morphological description of Clade Ⅱ (*L. ambustum* (Fr.) Singer, *L. anthracophilum* (Lasch) M. Lange & Sivertsen, and *L. atratum* (Fr.) Singer) and could not confirm the existence of this clade in China. The taxonomic treatments of Clade Ⅱ from China should be performed based on additional detailed investigations in later studies.

For a long time, only *O. lignatilis* was distinguished. *Ossicaulis lachnopus* was recognized several years after publishing its taxonomic treatment and invalid combination. In this study, *O. sichuanensis* clusters as a sister clade of *O. lachnopus*, *O. lignatilis*, and *O. yunnanensis* with strong support and can be easily distinguished by its morphology. The absence of cystidia and the unique ecology of *O. salomii* differs from other *Ossicaulis* species [45]. In the present paper, four new species of lyophylloid mushrooms are described from temperate and boreal China, among them *Lyophyllum atrofuscum*, *L. subalpinarum*, and *Ossicaulis sichuanensis* are from Southwest China, which demonstrates that Southwest China is very rich in the species diversity of fungi, as shown in previous studies [63,64,65,66,67]. A key to the 17 *Lyophyllum* species reported from China and the known species of *Ossicaulis* are provided as follows:
Key to species of *Lyophyllum* in China1. Lamellae black when bruised21’. Lamellae no staining when bruised132. Basidiocarps small, pileus usually less than 6 cm, stipe less than 5 cm, and width narrower than 1.2 cm3 2’. Basidiocarps medium to large, pileus usually more than 6 cm, stipe more than 5 cm long, and width exceeding 1.2 cm73. Lamellae dark, grayish-black*L. trigonosporum*3’. Lamellae white, gray to light brown44. Pileus usually less than 3 cm54’. Pileus usually broader than 3 cm65. Width of the stipe is narrower than 0.3 cm*L. pusillum*5’. Width of the stipe is usually 0.3–1.2 cm*L. pulvis-horrei*6. Pileipellis hyphae width of more than 4 μm *L. subalpinarum*6’. Pileipellis hyphae width less than or equal to 4 μm*L. semitale*7. Basidiospores usually longer than 14 μm*L. rhombisporum*7’. Basidiospores shorter than 14 µm88. Basidiospores globosae *L. immundum*8’. Basidiospores not globosae99. Basidiospores triangular109’. Basidiospores rhombic or subrhombic1110. Basidiospores with a hump located in the middle of the abaxial side, not thicker than the main body of the basidiospore and not higher than the length of the Basidiospore*L. sykosporum*10’. Basidiospores with a broad abaxial, thickening and making it widest near the apex*L. transforme*11. Q value less than or equal to 1.5*L. atrofuscum*11’. Q value higher than 1.51212. Hymenophoral trama are regular, hyphae of the mediostratum are narrow than 15 μm*L. infumatum*12’. Hymenophoral trama are regular, hyphae of the mediostratum exceed 15 μm, up to 20 μm*L. macrosporum*13. Basidiocarps large, up to exceeding 10 cm1413’. Basidiocarps are small to medium, less than 10 cm1514. Pileus reddish-brown to chestnut-brown, the width of stipe usually less than 1.5 cm*L. loricatum*14’. Pileus grayish-brown to grayish-yellow, the width of the stipe usually exceeds more than 1.5cm *L. shimeji*15. Basidiospores diam usually less than 5.5 μm*L. subdecastes*15’. Basidiospores diam usually more than 5.5 μm1616. Basidia usually less than 30 μm*L. fumosum*16’. Basidia usually longer than 30 μm*L. decastes*

Key to worldwide species of *Ossicaulis*1. Basidiospores usually shorter than 4 µm21’. Basidiospores usually longer than 4 μm32. Pileus white, chalky to orange tinged, without grayish tinge, on living tree trunks in alpine belt
*O. yunnanensis*
2’. Pileus gray or beige-gray, on dying or decaying woods
*O. lachnopus*
3. Width of the stipe is narrower than 2.0 cm
*O. salomii*
3’. Width of stipe is usually more than 2.0 cm44. Basidia usually less than 15 μm
*O. lignatilis*
4’. Basidia usually longer than 15 μm
*O. sichuanensis*


## Figures and Tables

**Table 1:** Taxa information and GenBank accession numbers of the sequences used in this study.

Taxa	Voucher ID	GenBank Accession No.		References
		ITS	nLSU	
*Asterophora lycoperdoides*	CBS170.86	AF357037	AF223190	[1]
*A. parasitica*	CBS683.82	AF357038	AF223191	[1]
*Calocybe carnea*	CBS552.50	AF357028	AF223178	[1]
*C. favrei*	IE-BSG-HC96cp4	EF421102	AF223184	[1]
*C. gangraenosa*	IE-BSG-HAe251.97	AF357032	AF223202	[1]
*C. ionides*	IE-BSG-HC77/133	AF357029	AF223179	[1]
*C. naucoria*	IE-BSG-HC80/103	AF357030	AF223180	[1]
*C. obscurissima*	IE-BSG-HC79/181	AF357031	AF223181	[1]
*C. ochracea*	IE-BSG-BSI94.cp1	AF357033	AF223185	[41]
*C. persicolor*	IE-BSG-HC80/99	AF357026	AF223176	[1]
*Clitolyophyllum akcaabatense*	KATO Fungi 3184	KT934393	KT934394	[42]
*Entoloma undatum*	TB7144	EF421108	AF261315	[1]
*Gerhardtia* sp.	HC01/025	EF421103	EF421091	[1]
*Hypsizygus ulmarius*	DUKE-JM/HW	EF421105	AF042584	[1]
*Leucocybe candicans*	AFTOL-ID 541	DQ202268	AY645055	[1]
*L. connata*	DUKE-JM90c	EF421104	AF042590	[1]
*Lyophyllum ambustum*	CBS452.87	AF357057	AF223216	[1]
*L. ambustum*	CBS450.87	AF357058	AF223214	[1]
*L. anthracophilum*	IE-BSG-HC79/132	AF357055	AF223212	[1]
*L. atratum*	CBS709.87	AF357053	AF223210	[1]
***L. atrofuscum***	**HMJAU63461**	**OP605493**	**OP605513**	**this study**
***L. atrofuscum***	**HMJAU63456**	**OP605494**	**OP605514**	**this study**
*L. caerulescens*	GC05082201	KP192628	NA	[3]
*L. caerulescens*	IE-BSG-HC80/140	AF357052	AF223209	[1]
*L.* cf. *helvella*	GC07101301	KP192625	NA	[3]
*L.* cf. *maas-geesterani*	PAM06082615	KP192553	NA	[3]
*L.* cf. *pulvis-horrei*	GC10101701	KP192665	NA	[3]
*L. crassipodium*	AB12-11-237	KP192608	NA	[3]
*L. decastes*	IE-BSG-JM87/16	AF357059	AF042583	[1]
*L. decastes*	Brunsson 19920924	HM572544	NA	[43]
*L. decastes*	Andersson 19901016	HM572546	NA	[43]
*L. decastes*	Bengtsson 19910929	HM572545	NA	[43]
*L. decastes*	LAS 06-152	HM572547	NA	[43]
*L. decastes*	Aase 19821031	HM572543	NA	[43]
*L. decastes*	Sundberg 20091007a	HM572548	NA	[43]
*L. decastes*	Sundberg 20091007b	HM572549	NA	[43]
*L. fumosum*	SJ 02-006	HM572539	NA	[43]
*L. fumosum*	Lipovac 20090903	HM572538	NA	[43]
*L. fumosum*	Sundberg 20090813	HM572537	NA	[43]
*L. infumatum*	AB04-11-497	KP192584	NA	[3]
*L. konradianum*	PAM06092203	KP192569	NA	[3]
*L. maleolens*	AB11-11-328	KP192607	NA	[3]
*L. maleolens*	GC08110109	KP192624	NA	[3]
*L. moncalvoanum*	PDD 72796	KJ461890	KJ461891	[44]
*L. moncalvoanum*	PDD 96328	KJ461904	KJ461905	[44]
*L. semitale*	CBS 369.47	AF357048	AF223207	[41]
*L. semitale*	IE-BSG-HC85/13	AF357049	AF042581	[1]
*L. shimeji*	Domeij090913	HM572525	NA	[43]
*L. shimeji*	Sundberg 20090813a	HM572524	NA	[43]
***L. subalpinarum***	**HMJAU63449**	**OP605490**	**OP605510**	**this study**
***L. subalpinarum***	**HMJAU63453**	**OP605491**	**OP605511**	**this study**
***L. subalpinarum***	**HMJAU63447**	**OP605492**	**OP605512**	**this study**
***L. subdecastes***	**HMJAU63470**	**OP605488**	**OP605508**	**this study**
***L. subdecastes***	**HMJAU63467**	**OP605489**	**OP605509**	**this study**
*L. sykosporum*	IE-BSG-HCM3	AF357051	AF357073	[41]
*L. sykosporum*	IFO30978	AF357050	AF223208	[1]
*L. transforme*	GC08101108	KP192653	NA	[3]
*Myochromella boudieri*	IE-BSG-HC78U	AF357046	AF223206	[41]
*M. inolens*	IE-BSG-BSI96/84	AF357047	AF223204	[1]
*M. inolens*	CBS330.85	AF357045	AF223201	[1]
*Ossicaulis lachnopus*	PRM 899221	HE649956	NA	[21]
*O. lachnopus*	PRM 899181	HE649955	NA	[21]
*O. lignatilis*	PRM 897367	HE649952	NA	[21]
*O. lignatilis*	PRM 889177	HE649953	NA	[21]
*O. salomii*	AB 14-04-02	MK650044	MK650043	[45]
***O. sichuanensis***	**HMJAU63481**	**OP605495**	**OP605515**	**this study**
***O. sichuanensis***	**HMJAU63482**	**OP605496**	**OP605516**	**this study**
***O. sichuanensis***	**HMJAU63483**	**OP605497**	**OP605517**	**this study**
*O. yunnanensis*	IJ152	KY411962	KY411960	[29]
*O. yunnanensis*	IH26	KY411961	KY411959	[29]
*Rhizocybe vermicularis*	AH44078	KJ681032	KJ681039	[46]
*Sagaranella gibberosa*	CBS328.50	AF357041	AF223197	[1]
*S. gibberosa*	CBS320.80/IFO 30977	AF357042	AF223198	[41]
*S. paluster*	CBS717.87	AF357044	AF223200	[1]
*S. tylicolor*	IE-BSG-BSI92/245	AF357040	AF223195	[1]
*S. tylicolor*	IE-BSG-Sag5-27/11	AF357039	AF223194	[41]
*S. paluster*	CBS714.87	AF357043	AF223199	[41]
*Tephrocybella griseonigrescens*	TO HG 21112014	KR105775	KR476785	[47]
*Tephroderma fuscopallens*	LUG18989	KJ701327	KJ701333	[47]
*T. fuscopallens*	EM4789-12	KJ701326	KJ701332	[47]
*Termitomyces heimii*	DUKE-JMleg.MUIDs.n.	AF357022	AF042586	[41]
*T. microcarpus*	DUKE-PRU3900	AF357023	AF042578	[1]
*T. radicatus*	CBS204.47	AF357025	AF223203	[1]
*T.* sp.	IE-BSG-BSI sp.1	AF357024	AF223174	[1]
*Tricholomella constricta*	IE-BSG-HC84/75	AF357036	AF223188	[1]
*Tricholyophyllum brunneum*	HKAS107494	MT705717	MT734655	[24]

Notes: Newly generated sequences in this study are in bold.

## Data Availability

Data relevant to this research can be found at https://www.ncbi.nlm.gov/, https://www.mycobank.org/, and https://www.treebase.org/treebase-web/home.html, accessed on 10 October 2022.
